# Function of Glia in Aging and the Brain Diseases

**DOI:** 10.7150/ijms.37769

**Published:** 2019-10-05

**Authors:** Soraya L. Valles, Antonio Iradi, Martin Aldasoro, Jose M. Vila, Constanza Aldasoro, Jack de la Torre, Juan Campos-Campos, Adrian Jorda

**Affiliations:** 1 Department of Physiology, School of Medicine, University of Valencia, Spain.; 2 University of Texas, Austin, Texas, USA.; 3 Department of Nursing, Faculty of Nursing and Podiatry, University of Valencia, Spain.

**Keywords:** Aging, brain diseases, glia.

## Abstract

Microglia cells during aging, neurodegeneration and neuroinflammation show different morphological and transcriptional profiles (related to axonal direction and cell adhesion). Furthermore, expressions of the receptors on the surface and actin formation compared to young are also different. This review delves into the role of glia during aging and the development of the diseases. The susceptibility of different regions of the brain to disease are linked to the overstimulation of signals related to the immune system during aging, as well as the damaging impact of these cascades on the functionality of different populations of microglia present in each region of the brain. Furthermore, a decrease in microglial phagocytosis has been related to many diseases and also has been detected during aging. In this paper we also describe the role of glia in different illness, such as AD, ALS, pain related disorders, cancer, developmental disorders and the problems produced by opening of the blood brain barrier. Future studies will clarify many points planted by this review.

## Microglia and Aging

The aged microglia of the human cerebral cortex to show different morphological abnormalities, including, spherical and knotty shapes, less branching, as well as fragmentation processes 1. Microglia isolated from post-mortem samples of parietal cortex in elderly people also show different transcriptional profiles from cells obtained in young subjects whose genes are associated with cell adhesion, axonal direction, receptor expression on their surfaces and actin formation where they were especially affected 2. Grabert and his group 3 found that changes at the genetic level related to the immune system, and to a lesser extent the genes related to cellular bioenergy, were widely associated with the diversity related to the brain region and its age 3.

Microglia are also greatly affected by aging and disease at the molecular level. Using "high-dimensional single-cell proteomic mapping" techniques, Mrdjen *et al.*, 4, identified diverse populations of inflammatory cells, including microglia, in the adult mouse brain. In the same way, it has also been possible to find remarkable changes and the proteomic signature of these cells during aging, neurodegeneration and neuroinflammation. Compared with cells from young mice, a population of aged microglia expresses higher levels of phagocytosis associated with the CD11c and CD14 markers. In addition, different populations of microglia were identified in the brains of old mice, where the reactive population expressed higher levels of CD11c and CD14, CD86, CD44, programming ligand of death 1 and MHC-II, and lower levels of markers of microglial homeostasis CX3CR1, MerTK (C-MER protooncogen tyrosine kinase), and Siglec-H, compared with non-reactive microglia 4. TNF-α, IL-1β, and IL-6 are among the proinflammatory cytokines that are overproduced during aging. Cribbs and his group 5 suspected that this was caused by overstimulation of the transcription factor NF-κB when the microglia underwent cell senescence 5. Biochemically, aged microglia produce more reactive oxidative species and inflammatory cytokines 6. In the same way, a positive relationship between these oxidative species and inflammatory cytokines has also been found in the brain and spinal cord of the APP/PS1KI mouse model of Alzheimer's disease 7.

The overproduction of proinflammatory mediators leads to microglia sensitization, or age-related microglial priming, so that aged microglia produce an exaggerated but inefficient response to inflammatory stimuli. Wendeln *et al*. 8 recently demonstrated that peripheral stimulation of mouse microglial cells by repeated injection of LPS can cause epigenetic changes in these cells for more than six months 8. This suggests that the basal level of inflammation in microglia can be increased with repetitive inflammatory stimuli, potentially being the cause of microglial priming. Cumulative and lasting changes in the balance of inflammatory mediators worsen the ability of microglia to perform basic physiological functions and are probably a contributing factor in neurodegenerative processes. For example, young microglia can more efficiently phagocytose disease-related proteins, such as Aβ 9 and α-synuclein 10 than aged microglia. Similarly, it has been shown that aged microglia decrease motility, vigilance, and phagocytic responses to demyelinating lesions 11, in part due to reduced lysosomal function 12. Taken together, these studies suggest that the susceptibility of different regions of the brain to Alzheimer's disease (AD) may be linked to the overstimulation of signals related to the immune system during aging, as well as the damaging impact of these cascades on the functionality of different populations of microglia present in each region of the brain.

## Decrease in Microglial Phagocytosis during Aging

As the main phagocytic cells in the brain, microglia play a central role in the clearance of Aβ. However, the effectiveness of this clearance decreases during aging, and particularly in AD 9. Despite its inability to clear Aβ from the brain, microglia continue to release proinflammatory mediators to further stimulate the immune response, thus creating a cycle that leads to the accumulation of activated immune cells, inflammatory mediators and Aβ. This cycle which is partially caused by microglia senescence has been reported by Thakker *et al.* 13 and can be reversed by blocking Aβ synthesis. 13. The microglia of old APP / PS1 mice exhibit lower expression of the scavenger receptor A (for its acronym in English SRA), CD36 and the receptor for RAGE (three scavenger receptors for beta-amyloid binding) when compared to that observed in the cells from young mice. By contrast, microglia from the group of APP / PS1 mice expresses higher levels of the proinflammatory cytokines, IL-1β and TNF-α, suggesting that there is an inverse correlation between the production of pro-inflammatory cytokines and clearance of Aβ. This idea is supported by *in vitro* studies in which treatment of microglia with TNF-α resulted in a reduction of the expression of SRA and CD36, and uptake of Aβ 14. In AD mouse models, microglia also show substantial deterioration in calcium signalling 15 and beclin-1 mediated recycling of phagocytic receptors CD36 and Trem2 16, which are linked to a poor internalization of Aβ.

Based on longitudinal studies of images of the human brain, Fan *et al.* indicated the state of microglia activation changes from an early protective phenotype to a late and noxious phenotype during the progression of AD 17. The chronic activation of different populations of microglia could be associated with the change in the global microglial phenotype depending on whether they are CX3CR1 + for the production of inflammatory mediators or Trem2 + for phagocytosis of Aβ 18.

Although there is strong evidence that aging impairs microglial activity, the relationship between microglial senescence, Aβ and AD remains incomplete, given that some studies have shown that microglial phagocytic activity towards Aβ is not necessarily related to changes in neurotoxicity and cognition. Investigations using the APP J20 mouse model have revealed that the inhibition of microglial phagocytic activity by minocycline before the accumulation of Aβ results in an increase in amyloid plaque burden, reduced inflammation, and improves cognitive performance, which indicates that chronic inflammation can interrupt normal neuronal function independent of Aβ. However, when microglial inhibition is performed after Aβ deposition begins, inflammation is suppressed by minocycline with no effect on amyloid plaque loading or in the improvement in cognitive performance 19. To add even more controversy to the relationship between inflammation and AD, the pharmacological or genetic suppression of microglia after Aβ accumulation does not change the plaque levels but prevents dendritic loss of the vertebral spine, neuronal loss and improves cognitive performance 20,21.

## Alzheimer's Disease and Glia

In Alzheimer's disease, complex changes and specific conflicts occur in different brain regions. The number of reactive astrocytes increases, engulfing and reducing the amyloid plaques. In addition, astrocytes surround the amyloid plaques and secrete proinflammatory factors in the extracellular space 22. Currently, no single hypothesis about what causes AD has been proven, a fact which argues for the heterogeneity of this dementia. For decades, it has been thought by many that the amyloid cascade hypothesis was the correct cause and this thinking was supported and promoted financially by a host of pharmaceutical companies around the world. The Aβ hypothesis argues that in AD there is an increase in oxidative stress caused by the accumulation of Aβ and that its elimination has been a priority but without positive results to the patients. There are much researches showing that increased levels of ROS have been linked to AD 23 but the effects of antioxidants in clinical studies have been disappointing, either because high concentrations of antioxidants are pro-oxidants, or because oxidative stress occurs relatively early in the course of the disease, or, because the combination of antioxidants fails in the clinical stage. Analogous to microglia, astrocytes play multiple roles in the organization and maintenance of brain structure and function. Multiple studies show that astrocytes dynamically modulate information processing, signal transmission, neural and synaptic plasticity, as well as homeostasis control of the blood-brain barrier. The astrocytic role in immune responses is not entirely clear. The evidence suggests that, astrocytes act as a protector during cerebral ischemia, whereas against inflammation mediated by the lipopolysaccharide of Escherichia coli, its intervention seems to be harmful 24. In the cells of the retina, however, it has been reported that through the production of lipoxins, astrocytes have an anti-inflammatory and neuroprotective effect against acute and chronic lesions 25. Similarly, the role of the cytokine IL-33 produced by astrocytes has recently been demonstrated to the microglial approach to the synaptic terminals, as well as the development of neural circuits 26. From prior studies discussed above on the action of IL-1α ^+^, it may be concluded that there is also a correlation between IL-1α and the greater number of GFAP ^+^ astrocytes (GFAP-immunoreactive astrocytes) 27. Conversely, it has been demonstrated in a study carried out in mice with multiple sclerosis that signals of the proinflammatory cytokine TNF-α promote in astrocytes a change in synaptic transmission and produce interference at the cognitive level 28. Other studies have shown that the activation of certain transcription factors are also involved in protective (STAT3) 29 or injurious (NF-κB) effects 30. More details about the relationships between glia cells and Alzheimer's disease can find them in 31.

## Amyotrophic Lateral Sclerosis (Als) and Glia

In ALS cortical and spinal motor neurons suffer damage, sequentially or simultaneously. Until 25 years ago, astrocytes were only considered the architecture of the CNS. Since then, many other functions have been described for these cells. The astrocytes adapt their function to the needs of neurons associated with them, and vary according to the mission they need to perform 32,33. The population of astrocytes connected to a neuron group have defined functions and morphological characteristics that are different from other astrocytic populations. Fibrous astrocytes have a fusiform body located in parallel with nerve fibers and produce extensions even directly on the nodes of Ranvier. 32,34. Each astrocyte controls a space with several motor neurons and makes contact with 3 to 10 neurons, hundreds of dendrites and with multiple synapses 35. Astrocytes connect to each other through gap-junctions (GJs). The GJs do the intercellular communication through ion exchange (electrical coupling) and the passage of small molecules (metabolic coupling) develops a type of syncytium that extends across the CNS. Therefore, all astrocytes in the brain constitute a network that helps neurons and other types of brain cells in the brain, controlling inflammation, oxidative stress, and brain nutrition 36. Furthermore, astrocytes control microglia and the inflammatory reaction produced by macrophages. The GJs communicate with protoplasmic and fibrous astrocytes, with neurons and their extensions as well as with arterioles 37. Motor neuron death, which occurs through mitochondrial, cytoplasmic apoptosis or vacuolization 38,39, can affect neurons by noxious circumstances. Astrocytes are also important because any alteration that affects cortical or spinal neurons will cause consequences in neurons far removed. However, astrocytes have different populations surrounding neuron types and that is true for protoplasmic astrocytes, which make contact with neurons, and for fibrous astrocytes that contact neuronal extensions 40,41. A recent paper, published by Larrodé *et al.* 42, demonstrated the involvement of DREAM (Downstream Regulatory Element Antagonist Modulator) in ALS. This protein could be used as a novel therapeutic target to treat ALS, but further studies are needed to understand the molecular mechanism of DREAM in ALS.

ALS begins within a group of cortical and/or spinal neurons, and protoplasmic astrocytes may send toxic mediators to other protoplasmic cells surrounding different neuronal groups. Furthermore, protoplasmic astrocytes are normally in communication with fibrous astrocytes connecting that with axons of the pyramidal cells 43. It is possible that these protoplasmic astrocytes could induce lethal damage in the cells implicated.

## Glia and Its Role in Pain

Pain plays a protective body role that functions as a signal of damage. Pain can convert into a disease when it persists more than 3 or 4 months (chronic pain) 44. In the sensory ganglia, particularly in the dorsal spinal ganglia, there are different types of cells. Cultures of the dorsal spinal ganglia in the rat demonstrate a presence of organized cells, seen using light and electron microscopy. These cells surround each body and the proximal portion of a neuronal axon forming a sheath around the cell. Each unit is a morphological and functional distinct body 45 and every unit is locked on with a neighbor by adhesion and gap junctions. Satellite glial (SG) cells are important in non-physiological pain and are a potential target for the development of new pain treatments 46. The cells in the sensory ganglia are surrounded by neuronal bodies presenting different functional units with bidirectional communication signaling between both types of neural cells. SG cells develop biochemical and structural changes after nerve damage, and consequently, the development of pain in animal models 47,48. A somatic release of chemical mediators, dependent on Ca^2+^, occurs after electric or chemical stimulation which alter somatic excitability in the sensory ganglia 49, such as substance P, glutamate, adenosine triphosphate, γ-amino-butyric acid and CGRP (calcitonin gene related peptide) 50,51.

The communication between cells in the sensory ganglia (SG), neuron-neuron; neuron-SG or SG-SG, can affect the communication between cells. The SG cells can modulate chronic pain 52,53 and sensory ganglia can be the pathophysiological first level of chronic pain. Furthermore, interaction between SG cells and neurons is becoming more and more important in the therapeutic field of chronic pain.

## Glia and Cancer

Tumor types are determined by age, size and location. In infancy and adolescence, infratentorial astrocytoma and midline tumors such as medulloblastoma and pinealoma predominate. In adulthood, anaplastic astrocytoma and glioblastoma are more common 54. Glioblastoma is the most common tumor detected in the brain, although the most frequent tumor seen at autopsy is meningioma. Secondary glioblastomas progress from low-grade diffuse astrocytoma or anaplastic astrocytoma 55. Some brain tumors, schwannoma, sarcoma, glioma and meningioma, persist after the patient has been exposed to chemotherapy and/or radiation therapy. In Epstein Barr virus and in primary brain lymphoma, virus infections are involved and in many cases, they are associated with AIDS.

Astrocytes are strongly associated with cancer in the brain 56 as cancerous astrocytoma. There are three different astrocytoma types, pilocytic, diffuse and anaplastic astrocytoma. Pilocytic astrocytoma is always treated by surgery and is more prevalent in children 57. In diffuse astrocytoma, surgery and radiotherapy is generally recommended. In anaplastic astrocytoma the same treatment as that for glioblastoma is used (indicated below) 58. Investigation of astrocytomas has been studied with the use of anti-cancer stem cell drugs 59. Another type of cancer is glioblastoma multiform which represents 20 % of all intracranial tumors, constituting half of all glioblastomas. They grow from tissue surrounding nerve cells and normally start in the cerebral hemispheric part of the brain. It affects 60% of men and 40% of women. They have high malignancy, producing quick invasion of the brain tissue 60. Because of their malignancy, natural and synthetic drugs have been used 61. Targeting cholesterol metabolism has also been used in cancer therapy to control changes in aberrant cholesterol production in neuroblastoma 62.

Non-neuron cells can develop cancer, such as astrocytes, and oligodendroglia. The neurons are differentiated cells, so they cannot produce cancer. The neuroblastoma cells are radial glia or precursors of astrocytes that can develop before their differentiation to neurons. Glial cells develop different types of cells such as neurons, oligodendroglia and astrocytes 63. All these types of cells can convert to cancer and affect the normal function of the brain). Radial glia are stem cells that develop from a progenitor stem cell in the embryo and in the adult brain 64. It is of interest to point out that in some astrocytomas, the patient may increase cognitive capacity and memory, before the illness starts and even when cancer is already present 65.

## Glia and Developmental Disorders

Astrocytes play a role in the aberrant formation of neuronal circuitry in the brain. This field in neuroscience is relatively new and shows us the involvement of astrocytes in psychiatric disorders, including autism spectrum disorders (ASDs), bipolar disorders (BD) and schizophrenia 66,67. The expression of the MicroRNAs are critical in the development of prefrontal cortical circuitry during the postnatal brain development. Deficits in neuronal maturation produced by the microRNAs decrease and/or become deficient and can be demonstrated in some neuropsychiatric diseases 67. Coordination of the different neural cell types is fundamental in the development of the neural network and in normal neural function. Astrocytes coordinate neural development by controlling the formation of synapses, neuronal function, driving axon growth, and promoting neuronal survival 68. Astrogenesis occurs after neurons develop in the brain of rats 64, and in other animals, including humans 69. During development of the brain, chromatin changes occur and defects in astrogenesis or in early function of astrocytes that can produce the progression of neurodevelopmental disorders 68.

## The Opening of the Blood-Brain Barrier during Aging

Another characteristic of aging with relevance for AD is the increase in the permeability of the blood-brain barrier (BBB) ​​to immune cells and the peripheral tissue molecules. The loss of integrity of the BBB appears to occur prior to hippocampal atrophy 70,71 and cognitive impairment 72, suggesting that this decomposition precedes the neurodegenerative process in AD. This evidence also indicates that the peripheral activation of the immune system may contribute to the deterioration of brain function and the neurodegenerative processes that occur in AD 73. Interestingly, an association has recently been made between inflammation in middle-aged individuals and brain volume in older individuals. Compared with middle-aged people without elevated inflammatory markers, individuals with elevations in 3 or more markers had, on average, 5% more decreased of the volume hippocampus, typical of the AD. 71. In healthy individuals, those responsible for the proper functioning of neurovascular function are the pericytes, endothelial cells and astrocytes, which form fine junctions between endothelial cells in response to inflammation that limit the consequent mobilization of immune cells from the periphery. It has been found that inhibiting the formation of the inducible astrocytic barrier increases the severity of the disease in mouse models. In this way, it is suspected that the senescence of the cellular components that form this barrier is one of the causes behind the malfunction of the BBB 74.

Endothelial cells, pericytes and astrocytes in the BBB are particularly vulnerable to the effects of aging and chronic stimulation by inflammatory mediators. During aging, endothelial cells in the mouse brain express higher levels of TNF-α and decreased expression of the narrow-binding proteins ocludin-1 and zonula occludens1, which correlate with an increase in peripheral inflammation 75. Aging and, more aggressively, AD, also cause damage to the pericytes as evidenced by the increase in β-platelet-derived growth factor receptor levels observed in experiments performed on mice 70. Although the mechanism of induction of pericyte lesions is still unknown, Bell and his collaborators have shown that age-dependent vascular damage in pericyte-deficient mice precedes neuronal degenerative changes, learning and memory impairment, and neuroinflammatory response 76.

In astrocytes, transcriptome analysis has revealed that aging induces upregulation of several genes related to the immune system 77. An age-dependent change in the phenotype of the astrocytes was identified by comparing gene expression in the astrocytes of 10-week and 2-year-old mice using RNAseq. It has been demonstrated that IL-1β suppresses the astrocytic expression of "sonic hedgehog" 78, a protein that protects the BBB by upregulation of the tight binding proteins in capillary endothelial cells 79. Similarly, this study demonstrated that astrocytes from healthy 2-year-old mice expressed genetic markers that correspond to the activated A1 phenotype, including C4a, C3, Serpina3n, and Cxcl10 80. IL-1β also increases the production of proinflammatory chemokines such as CCL2, CCL20 and CXCL2 by astrocytes, which induces the migration of immune cells from the periphery and exacerbates BHE malfunction and neuroinflammation 78. Therefore, an excessive proinflammatory phenotype significantly alters the protective role of astrocytes in the maintenance of BBB integrity.

Inflammation and aging are therefore closely linked thanks to studies suggesting that low levels of inflammation correlate better with healthy brain function 71 and longevity 81. Considering that most cells in the brain, including astrocytes 30 and microglia 82, have a long lifespan, it is plausible that the accumulation and overstimulation of Inflammation can trigger multiple cumulative molecular modifications (telomere shortening, DNA damage, epigenetic modifications, lysosomal dysregulation) that eventually contribute to cellular senescence and loss of function. This idea is, at least in part, supported by a repopulation study in a neurodegeneration model of mice, which showed that, following a pharmacologically induced cell depletion, the microglia that repopulated the brain showed the morphological phenotype of young cells. Surprisingly, the animals also showed a significant improvement in brain function 83. It is still unknown that cell repopulation methods can restore the molecular signatures of immunesenescence. Additional studies are needed to clarify the underlying cellular and molecular mechanisms related to the immune dysregulation that occurs during aging that divert individuals from the relatively benign process of normal brain aging to the pathological processes associated with AD.

## Conclusion

The functions that the glia develops, both during the age and the illness, is an important field of study at present. The most important brain cells can be divided into neurons and glia. For many years research has focused on neurons, but increasingly the glia has becoming more important. Within the glia are the astrocytes, oligodendroglia and microglia. Many diseases affect one or several of these cells. From Alzheimer's disease, amyotrophic lateral sclerosis, pain-related diseases, cancer and developmental disorders, we know now that glia play an important role in the development of their diseases. Studies are needed to clarify the underlying cellular and molecular mechanisms related to the immune dysregulation that occurs during aging that divert individuals from the relatively benign process of normal brain aging to the pathological processes associated with different diseases.
